# Pacemaker Status and 5‐Year Mortality After TAVI: A Sex‐Specific Analysis

**DOI:** 10.1111/eci.70242

**Published:** 2026-07-02

**Authors:** Cecilia Veraar, Gudrun Lamm, Maximilian Will, Matthias Hammerer, Matthias Granner, Lion Merl, Konstantin Schwarz, Julia Mascherbauer

**Affiliations:** ^1^ Department of Internal Medicine 3 University Hospital St. Pölten – NOE LGA, Karl Landsteiner University St. Pölten Austria; ^2^ Department of Internal Medicine II Paracelsus Medical University of Salzburg Salzburg Austria

**Keywords:** aortic stenosis, mortality, pacemaker, sex differences, transcatheter aortic valve implantation

## Abstract

**Background:**

Sex‐specific differences regarding the prognostic impact of permanent pacemaker (PM) implantation after transcatheter aortic valve implantation (TAVI) remain largely unexplored.

**Objectives:**

To investigate sex‐specific associations between pre‐existing and new post‐procedural PM implantation and short‐ and long‐term mortality after TAVI.

**Methods:**

This post hoc analysis of a prospective registry included consecutive patients who underwent TAVI between 2016 and 2020. Patients were stratified by sex and assigned to three groups: no PM, pre‐existing PM (pre‐PM) and PM implantation within 30 days of TAVI (post‐PM). The primary endpoint was 5‐year all‐cause mortality; the secondary endpoint was 30‐day mortality. Sex‐stratified multivariable proportional hazards models and Kaplan–Meier analyses were performed.

**Results:**

Among 1114 patients (81 ± 5 years, 49.8% female), women more frequently had no PM (80.2% vs. 70.8%, *p* < 0.001) and less often had a pre‐existing PM (7.0% vs. 14.5%, *p* < 0.001). Post‐PM rates were similar between sexes (12.8% vs. 14.7%, *p* = 0.172). Thirty‐day survival differed by PM status only in women, with worse outcomes in the post‐PM group (log‐rank *p* = 0.033), whereas no difference was observed in men (*p* = 0.542). Five‐year survival did not differ by PM status among women (*p* = 0.572) but differed significantly among men (*p* < 0.001), with the lowest survival in men with pre‐PM.

**Conclusion:**

Post‐procedural PM implantation was associated with higher 30‐day mortality in women, whereas pre‐existing PM predicted reduced 5‐year survival in men, supporting sex‐specific risk assessment before and after TAVI.

## Graphical abstract

Among patients undergoing TAVI, the prognostic impact of pacemaker status differed substantially between women and men. Women receiving a new pacemaker after TAVI exhibited higher 30‐day mortality, suggesting increased vulnerability to procedural complications; however, long‐term survival was unaffected. In contrast, men with a pre‐existing pacemaker had significantly worse 5‐year survival. These findings suggest that acute procedural factors predominate in women, whereas underlying conduction disease appears to be a major determinant of long‐term prognosis in men.

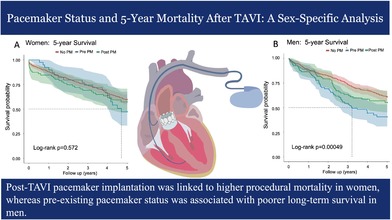

## 1 Introduction

Transcatheter aortic valve implantation (TAVI) has become an established treatment for symptomatic severe aortic stenosis, with current guidelines recommending TAVI for patients aged ≥ 70 years who are suitable for transfemoral access [1]. As indications expand toward younger, lower‐risk populations, the potential long‐term consequences of permanent pacemaker (PM) implantation are gaining importance in treatment decisions and patient counselling [2].

Women and men with aortic stenosis differ substantially in baseline anatomy, comorbidity profiles and post‐procedural outcomes. Women typically present with smaller annular dimensions, less coronary artery disease and higher prevalence of paradoxical low‐flow/low‐gradient aortic stenosis [3, 4, 5], whereas men more frequently exhibit pre‐existing conduction disturbances and impaired left ventricular systolic function [4]. Although large registries suggest that women may achieve similar or superior long‐term survival after TAVI compared with men, results remain heterogeneous, indicating that additional sex‐specific factors influence prognosis [6, 7]. PM implantation is among the most common TAVI‐related complications, occurring in up to 25% of patients depending on prosthesis design and baseline conduction abnormalities [8, 9]. Right ventricular pacing has been associated with adverse ventricular remodelling, pacing‐induced dyssynchrony and potentially impaired long‐term survival [10, 11]. Previous studies have predominantly assessed the overall impact of PM implantation on TAVI outcomes, but sex‐specific differences remain poorly understood. In particular, whether the prognostic impact of pre‐existing PM (pre‐PM, reflecting chronic conduction disease) differs from new post‐procedural PM (post‐PM, indicating acute conduction disturbances) in women and men remains unknown.

This study investigated 30‐day and 5‐year all‐cause mortality after TAVI stratified by sex and PM status, distinguishing pre‐existing from new post‐procedural PM implantation and comparing both with patients without PM.

## 2 Methods

### 2.1 Study Design and Population

This post hoc analysis of a prospective, single‐center registry included all consecutive patients undergoing TAVI at the Department of Cardiology, University Hospital St. Pölten, Austria, between January 1, 2016 and December 31, 2020. Long‐term vital status was obtained through national records (Statistik Austria) through December 31, 2023. While follow‐up extended up to 8 years, completeness decreased beyond 5 years. To minimize informative censoring and ensure consistent follow‐up across PM groups and sexes, 5‐year all‐cause mortality was defined as the primary endpoint.

Patients were stratified by sex and categorized into three PM groups based on VARC‐2 definitions:

1. No‐PM: no PM before TAVI and no PM implantation within 30 days post‐TAVI.
2. Pre‐PM: PM implanted before TAVI.
3. Post‐PM: new PM implanted within 30 days post‐TAVI.

PM implantations beyond 30 days, though potentially reflecting delayed TAVI‐related conduction injury, were classified as no‐PM in accordance with VARC definitions to maintain mechanistic specificity for procedure‐related events. Patients with an implantable cardioverter‐defibrillator (ICD), with or without cardiac resynchronization therapy (CRT), were included in the primary analysis according to their PM status. Sensitivity analyses were subsequently performed after exclusion of these patients.

### 2.2 Ethical Approval

The study was conducted in accordance with the Declaration of Helsinki (2013 revision) and approved by the Ethics Committee of the Karl Landsteiner University of Health Sciences (EK1066/2022). Informed consent was waived because of the anonymized registry design and the use of routinely collected clinical data.

### 2.3 Procedural Management, Follow‐Up and Data Collection

Patient eligibility for TAVI was determined by a multidisciplinary heart team. Device selection was at the operator's discretion. Temporary PMs were placed in all patients without a prior PM or ICD and removed if permanent pacing was not required. Continuous rhythm monitoring consisted of 72‐h telemetry, baseline and daily 12‐lead ECGs during post‐TAVI hospitalization, and systematic documentation of new conduction abnormalities. Permanent PM implantation followed ESC pacing guideline recommendations [12]. The institutional registry provided comprehensive demographic, clinical, laboratory, ECG and echocardiographic data collected pre‐procedurally. A structured transthoracic echocardiogram was performed before discharge according to the institutional protocol including standardized assessment of prosthesis function, transvalvular gradients, paravalvular regurgitation and left ventricular function. Chronic kidney disease (CKD) was recorded as a pre‐existing comorbidity based on the patient's documented medical history at the time of TAVI evaluation.

### 2.4 Endpoints

The primary endpoint was 5‐year all‐cause mortality, examined separately in women and men. The secondary endpoint comprised 30‐day all‐cause mortality.

### 2.5 Statistical Analysis

Continuous variables were reported as mean ± standard deviation (SD) and compared across groups using analysis of variance (ANOVA). Pairwise comparisons between pacemaker groups were adjusted using the Bonferroni method. Categorical variables were presented as counts and percentages and compared using chi‐square tests.

Sex‐specific determinants of permanent PM implantation were assessed using univariate and multivariable logistic regression models constructed separately for women and men. Variables with *p* < 0.05 in univariate analysis were included in multivariable models.

For 5‐year mortality, sex‐stratified Cox proportional hazards models were applied. The proportional hazards assumption was evaluated using Schoenfeld residuals.

Continuous variables were dichotomized using median cutoffs derived from the entire cohort (men and women combined) to ensure uniform thresholds across sexes. Median‐based categorization was chosen to facilitate clinically interpretable effect estimates, improve model stability in sex‐stratified analyses with limited event numbers, and allow direct comparison of risk estimates between women and men using identical thresholds while minimizing the risk of model overfitting. Median‐based categorization was chosen after exploratory analyses demonstrated non‐linear risk relationships and violations of proportional hazards assumptions. This approach improved model stability in sex‐stratified analyses and yielded clinically interpretable effect estimates while avoiding overfitting. Kaplan–Meier survival curves were generated for the three PM groups within each sex, with annual survival probabilities and numbers at risk. Between‐group differences were assessed using the log‐rank test. No variable exceeded 5% missingness; therefore, complete‐case analysis was performed.

All analyses were conducted using R version 4.4.1 (R Foundation for Statistical Computing, Vienna, Austria).

## 3 Results

A total of 1114 patients were included, of whom 555 (49.8%; mean age 81.6 ± 5.3 years) were women and 559 (50.2%; mean age 80.4 ± 6.1 years) were men. Women were less likely to require PM implantation at any time point (80.2% in women vs. 70.8% in men, *p* < 0.001), and men more often carried a PM prior to TAVI (7.0% in women vs. 14.5% in men; *p* < 0.001).

Among patients requiring post‐TAVI PM implantation, the most common indications were high‐grade atrioventricular block (47.1%), complete atrioventricular block (30.4%) and new‐onset left bundle branch block (11.0%), while the remaining 11.5% were implanted for other conduction abnormalities or bradyarrhythmic indications.

The rate of implantable cardioverter‐defibrillator (ICD) with and without cardiac resynchronization therapy (CRT) was low (0.4% women vs. 1.8% men, *p* = 0.021).

Periprocedural complications differed between sexes. Significant bleeding occurred more frequently in women than in men (3.1% vs. 0.7%, *p* = 0.001). Rates of transient ischemic attack were low and comparable between sexes (0.7% vs. 0.4%, *p* = 0.408). Women also showed numerically higher rates of stroke within 72 h after TAVI (3.1% vs. 1.4%, *p* = 0.066), cardiac tamponade (2.5% vs. 1.1%, *p* = 0.069) and cardiopulmonary resuscitation (0.5% vs. 0.0%, *p* = 0.082), although these differences did not reach statistical significance. Further details on early periprocedural complications are provided in Table S1.

### 3.1 Baseline Clinical and Demographic Characteristics

Baseline characteristics stratified by sex and PM status (Table 1A,B) revealed distinct patterns.

**TABLE 1:** Baseline clinical and demographic characteristics stratified by sex and PM status.

A. Women							
	No PM (1)	Pre‐PM (2)	Post‐ PM (3)	ANOVA/chi^2^	1–2	1–3	2–3
	*n* = 445	*n* = 38	*n* = 71	*p* (overall)	*p*	*p*	*p*
**Demographic data**							
Age^b^	81.4 ± 5.2	82.5 ± 5.6	82.0 ± 5.6	0.332	0.597	1.000	1.000
BMI^b^	28.3 ± 9.3	27.9 ± 6.4	28.9 ± 14.0	0.863	1.000	1.000	1.000
**Comorbidities and biomarkers**							
EuroSCORE II^b^	4.9 ± 5.1	5.84 ± 3.5	5.4 ± 5.6	0.459	0.879	1.000	1.000
AF^a^	146 (32.7)	19 (51.4)	25 (35.2)	0.121			
CAD^a^	186 (41.7)	15 (40.5)	27 (38.0)	0.790			
Diabetes^a^	147 (33.0)	12 (32.4)	22 (31.0)	0.539			
PVD^b^	22 (4.9)	4 (10.8)	6 (8.5)	0.231			
Troponin^b^	30.1 ± 37.0	25.9 ± 13.6	25.5 ± 14.4	0.500	1.000	0.950	1.000
NT‐proBNP^b^	3134 ± 5811	5591 ± 10,266	2529 ± 4435	**0.047**	0.069	1.000	0.053
Creatinine^b^	1.1 ± 0.4	1.2 ± 0.4	1.1 ± 0.3	0.346	0.508	1.000	1.000
CRP^b^	0.7 ± 1.0	0.7 ± 1.1	0.40 ± 0.52	0.231	1.000	0.288	0.489
**ECG‐parameters (prior to intervention)**							
QRS^b^	96.6 ± 21.9	117.3 ± 30.1	108.6 ± 27.7	**< 0.001**	**< 0.001**	**< 0.001**	0.361
PQ interval^b^	177.5 ± 91.3	188.8 ± 43.3	170.2 ± 39.0	0.718	1.000	1.000	1.000
Heart rate^b^	74.0 ± 15.1	66.3 ± 11.5	72.4 ± 14.0	**0.008**	**0.006**	1.000	0.125
**Echo‐parameters (prior to intervention)**							
LVEF^b^	56.0 ± 9.0	53.1 ± 10.2	57.3 ± 7.9	0.063	0.151	0.825	0.058
Mean gradient^b^	47.8 ± 15.3	45.6 ± 18.2	49.2 ± 15.3	0.527	1.000	1.000	0.775
AVA^b^	0.6 ± 0.1	0.6 ± 0.1	0.71 ± 0.1	0.065	1.000	0.106	0.133
AV Vmax^b^	4.2 ± 0.7	4.1 ± 0.9	4.2 ± 0.7	0.712	1.000	1.000	1.000
Valve type							
BEV^a^	330 (81.9)	25 (6.2)	47 (11.7)	0.621			
SEV^a^	92 (76.0)	11 (9.1)	18 (14.9)				
MEV^a^	24 (77.4)	1 (3.2)	6 (19.4)				

B. Men							
	No PM (1)	Pre‐PM (2)	Post‐ PM (3)	ANOVA/chi^2^	1–2	1–3	2–3
	*n* = 396	*n* = 81	*N* = 82	*p* (overall)	*p*	*p*	*p*
**Demographic data**							
Age^b^	79.6 ± 6.0	82.4 ± 6.0	81.9 ± 5.5	**< 0.001**	**< 0.001**	**0.006**	1.000
BMI^b^	27.6 ± 4.6	28.8 ± 12.3	28.2 ± 11.6	0.441	0.675	1.000	1.000
**Comorbidities and biomarker**							
AF^a^	164 (41.4)	41 (57.7)	30 (36.6)	**0.040**			
CAD^a^	237 (59.8)	42 (59.2)	44 (53.7)	0.663			
Diabetes^a^	137 (34.6)	22 (31.0)	21 (25.6)	0.449			
PVD^a^	32 (8.1)	9 (12.7)	10 (12.2)	0.457			
EuroSCORE II^b^	4.8 ± 5.9	6.0 ± 6.0	5.4 ± 6.2	0.201	0.274	1.000	1.000
Troponin^b^	40.3 ± 43.3	56.7 ± 122.9	44.8 ± 33.4	0.127	0.130	1.000	0.775
NT‐proBNP^b^	4381 ± 8731	5510 ± 9152	3125 ± 4745	0.245	0.908	0.788	0.282
Creatinine^b^	1.3 ± 0.8	1.3 ± 0.6	1.3 ± 0.5	0.855	1.000	1.000	1.000
CRP^b^	0.8 ± 1.8	0.5 ± 0.7	0.9 ± 2.2	0.457	0.862	1.000	0.787
**ECG‐parameters (prior to intervention)**							
QRS^b^	106.9 ± 25.2	132.1 ± 33.9	120.1 ± 25.4	**< 0.001**	**< 0.001**	**< 0.001**	0.085
PQ interval^b^	185.9 ± 44.4	201.1 ± 58.7	208.8 ± 53.6	**0.004**	0.562	**0.005**	1.000
Heart rate^b^	71.8 ± 13.4	66.0 ± 10.3	69.5 ± 12.1	**< 0.001**	**< 0.001**	1.000	0.258
**Echo‐parameters (prior to intervention)**							
LVEF^b^	51.5 ± 12.1	46.9 ± 13.5	54.1 ± 10.2	**< 0.001**	**0.006**	0.202	**< 0.001**
Mean gradient^b^	44.2 ± 14.9	37.3 ± 13.3	43.0 ± 12.8	**< 0.001**	**< 0.001**	1.000	**0.040**
AVA^b^	0.7 ± 0.1	0.7 ± 0.1	0.7 ± 0.1	0.231	0.474	0.698	1.000
AV Vmax^b^	4.0 ± 0.7	3.8 ± 0.7	4.0 ± 0.6	**0.049**	**0.043**	1.000	0.321
**Valve type**							
BEV^a^	376 (71.8)	65 (12.4)	74 (14.1)	0.621			
SEV^a^	13 (56.5)	4 (17.4)	5 (21.7)				
MEV^a^	7 (58.3)	2 (16.7)	3 (25.0)				

*Note:* This table summarizes clinical, demographic, laboratory, ECG and echocardiographic characteristics across the no‐PM, pre‐PM and post‐PM groups in women and men. Bold levels are significant.

Abbreviations: AF, atrial fibrillation; AV, aortic valve; AVA, aortic valve area; BEV, balloon‐expandable valve; BMI, body mass index; CAD, coronary artery disease; CRP, C‐reactive protein; DM, diabetes mellitus; LAH, left anterior hemiblock; LBBB, left bundle branch block; LVEF, left ventricular ejection fraction; MEV, mechanical expandable valve; No PM, no pacemaker neither before nor within 30 days after TAVI; NT‐proBNP, N‐terminal pro‐brain natriuretic peptide; PAD, peripheral artery disease; Post‐PM, new pacemaker after TAVI; Pre‐PM, pre‐existing pacemaker; RBBB, right bundle branch block; SEV, self‐expandable valve, THV, transcatheter heart valve; TF, transfemoral; Vmax, maximum velocity.

^a^ *n* (%).

^b^ Mean ± SD.

#### 3.1.1 Women

Women showed no statistically significant differences across groups regarding age, body mass index (BMI) or major comorbidities including atrial fibrillation (AF), coronary artery disease (CAD), diabetes mellitus [13], peripheral artery disease [14] and CKD. Differences were primarily electrocardiographic: pre‐PM and post‐PM women had longer QRS durations than no‐PM women (96.6 ± 21.9 vs. 117.3 ± 30.1 vs. 108.6 ± 27.7 ms, *p* < 0.001), and pre‐PM women showed lower heart rates compared no‐PM women (66.3 ± 11.5 vs. 74.0 ± 15.1 bpm, *p* = 0.006). NT‐proBNP levels were highest in pre‐PM vs. post‐ and no‐PM women (5591 ± 10,266 vs. 3134 ± 5811 vs. 2529 ± 4435 pg/mL, *p* = 0.047), whereas echocardiographic parameters were similar across groups.

#### 3.1.2 Men

In men, baseline differences between PM groups were more pronounced. Pre‐PM and post‐PM men were older (82.4 ± 6.0 and 81.9 ± 5.5 vs. 79.6 ± 6.0 years, *p* < 0.001), with AF more prevalent in pre‐PM patients (57.7% vs. 41.4%, *p* = 0.040). QRS duration was prolonged in both pre‐PM and post‐PM men versus no‐PM (106.9 ± 25.2 vs. 132.1 ± 33.9 vs. 120.1 ± 25.4 ms, *p* < 0.001), and the PQ interval was longer in post‐PM men (208.8 ± 53.6 vs. 185.9 ± 44.4 ms, *p* = 0.004). Pre‐PM men had lower LVEF (46.9% ± 13.5% vs. 51.5% ± 12.1%, *p* = 0.006) and lower mean gradients (37.3 ± 13.3 vs. 44.2 ± 14.9 mmHg, *p* < 0.001), while aortic valve area and peak jet velocity were comparable across groups.

### 3.2 Sex‐Specific Predictors of Pacemaker Requirement After TAVI

Conduction disturbances showed the strongest and most consistent associations with PM implantation across both sexes, though additional clinical and anatomical predictors differed by sex (Table 2A,B).

**TABLE 2:** Sex‐specific logistic regression models for predictors of PM implantation after TAVI.

	Univariate			Multivariate		
	OR	CI 95%	*p*	OR	CI 95%	*p*
**A: Women**						
Age > 82 years	1.0	0.7–1.6	0.841			
CKD	2.1	0.5–6.7	0.245			
DM	0.9	0.6–1.5	0.808			
Stroke	0.6	0.2–1.2	0.189			
Porcelain aorta	**2.6**	1.2–5.7	**0.015**	**2.8**	1.2–6.2	**0.014**
CAD	0.9	0.6–1.4	0.797			
Euroscore II > 5 points	**1.6**	1.0–2.4	**0.047**	1.2	0.7–1.9	0.521
NT‐proBNP ≥ 1532 ng/L	1.2	0.8–1.8	0.480			
Troponin > 24 ng/mL	1.0	0.6–1.6	0.974			
Creatinine ≥ 1.1 mg/dL	1.7	1.1–2.5	**0.019**	1.5	0.9–2.4	0.103
EF > 60%	1.0	0.6–1.6	0.873			
AVA < 0.7 cm^2^	0.8	0.5–1.2	0.205			
AF	1.4	0.9–2.1	0.156			
LBBB	0.3	0.1–0.8	0.043	0.3	0.1–1.0	0.074
RBBB	**8.2**	3.8–18.5	**< 0.001**	**8.1**	3.7–18.6	**< 0.001**
Prosthesis size > 26 mm	**1.7**	1.0–2.7	**0.041**	**1.7**	1–2.9	**0.034**
Balloon pre‐dilatation	1.4	0.4–4.0	0.599			
Balloon post‐dilatation	0.9	0.5–1.6	0.802			
**B: Men**						
Age > 82 years	**2.1**	1.5–3.1	**< 0.001**	**2.1**	1.3–3.2	**0.001**
CKD	0.9	0.3–2.1	0.797			
DM	0.8	0.5–1.2	0.288			
Stroke	1.4	0.8–2.3	0.194			
Porcelain aorta	0.9	0.3–2.5	0.896			
CAD	0.9	0.6–1.3	0.674			
Euroscore II > 5 points	1.4	0.9–2.1	0.086			
NT‐proBNP ≥ 1532 ng/L	1.0	0.7–1.5	0.910			
Troponin > 24 ng/mL	**2.1**	1.3–3.3	**0.002**	**2.0**	1.2–3.3	**0.008**
Creatinine ≥ 1.1 mg/dl	1.4	1–2.1	0.061			
EF > 60%	0.9	0.6–1.3	0.610			
AVA < 0.7 cm^2^	**0.6**	0.4–1	**0.038**	**0.6**	0.3–0.9	**0.027**
AF	1.2	0.8–1.7	0.346			
LBBB	0.5	0.2–1	**0.054**			
RBBB	**4.7**	2.5–9.2	**< 0.001**	**3.7**	1.8–7.9	**< 0.001**
Prosthesis size > 26 mm	**1.8**	1.2–2.6	**0.004**	**1.7**	1.1–2.7	**0.024**
RapidPacing	0.6	0.1–1.9	0.434			
Balloon pre‐dilatation	0.3	0–1.7	0.257			
Balloon post‐dilatation	1.3	0.5–2.9	0.532			

*Note:* This table presents univariable and multivariable predictors of PM implantation analysed separately for women and men. Bold levels are significant.

Abbreviations: AF, atrial fibrillation; CAD, coronary artery disease; CI, confidence interval; DM, diabetes mellitus; LAH, left anterior hemiblock; LBBB, left bundle branch block; LVEF, left ventricular ejection fraction; NT‐proBNP, N‐terminal pro‐brain natriuretic peptide; OR, Odds ratio; PAD, peripheral artery disease; RBBB, right bundle branch block.

In women, RBBB (OR 8.1, *p* < 0.001), porcelain aorta (OR 2.8, *p* = 0.014), and prosthesis size > 26 mm (OR 1.7, *p* = 0.034) remained independent predictors of post‐PM on multivariable analysis, while LBBB and creatinine lost significance after adjustment. In men, age > 82 years (OR 2.1, *p* = 0.001), troponin T serum levels (OR 2.0, *p* = 0.008), AVA < 0.7 cm^2^ (OR 0.6, *p* = 0.027), prosthesis size > 26 mm (OR 1.7, *p* = 0.024) and RBBB (OR 3.7, *p* < 0.001) remained independent predictors on multivariable analysis.

### 3.3 Sex‐Specific Association Between Pacemaker Status and Mortality After TAVI

The relationship between PM status and mortality after TAVI showed distinct sex‐specific patterns as depicted in Table 3A,B.

**TABLE 3:** Sex‐stratified Cox proportional hazards models for 5‐year all‐cause mortality.

Variable	Univariate			Multivariate		
	HR	CI 95%	*p*	HR	CI 95%	*p*
**A: Women**						
Pre‐PM	1.2	0.7–2.2	0.350			
Post‐PM	1.1	0.7–1.6	0.551			
Age > 82 years	**1.4**	1.1–1.8	**0.014**	**1.5**	1.0–2.1	**0.030**
CKD	**2.3**	1.2–4.5	**0.015**	**12.0**	1.5–92.9	**0.018**
DM	1.2	0.9–1.6	0.196			
Stroke	1.2	0.8–1.9	0.398			
Porcelain aorta	**1.8**	1.1–3.0	**0.016**	1.8	1.0–3.3	0.068
CAD	1.1	0.8–1.4	0.624			
Euroscore II > 5 points	**1.6**	1.2–2.1	**< 0.001**	1.1	0.7–1.6	0.772
NT‐proBNP ≥ 1532 ng/L	**2.1**	1.6–2.9	**< 0.001**	**1.8**	1.2–2.6	**0.003**
Troponin > 24 ng/ml	**1.9**	1.4–2.5	**< 0.001**	**1.5**	1.1–2.2	**0.019**
Creatinine ≥ 1.1 mg/dl	**1.7**	1.3–2.2	**< 0.001**	**1.5**	1.1–2.1	**0.024**
Mean gradient > 43 mmHg	**0.6**	0.5–0.8	**< 0.001**	**0.7**	0.5–1.0	**0.043**
EF > 60%	**0.7**	0.5–1.0	**0.043**	1.0	0.7–1.6	0.845
AVA < 0.7 cm^2^	1.1	0.8–1.4	0.702			
Stroke post TAVI	1.4	0.7–2.8	0.360			
AF	**1.9**	1.4–2.4	**< 0.001**	**1.8**	1.3–2.4	**< 0.001**
LBBB	1.3	0.8–2.0	0.302			
RBBB	1.4	0.9–2.4	0.176			
RapidPacing	1.4	0.7–2.6	0.312			
Balloon pre‐dilatation	1.6	0.8–3.2	0.147			
Balloon post‐dilatation	1.2	0.9–1.7	0.256			
Prosthesis size > 26 mm	1.1	0.8–1.6	0.457			
**B: Men**						
Pre‐PM	**1.8**	1.3–2.6	**< 0.001**	**1.7**	1.1–2.5	**0.011**
Post‐PM	1.2	0.8–1.8	0.191	1.1	0.6–1.7	0.681
Age > 82 years	**1.4**	1.1–1.8	**0.011**	1.2	0.8–1.7	0.257
CKD	**2.1**	1.3–3.5	**0.002**	**2.3**	1.2–4.7	**0.012**
DM	1.1	0.8–1.4	0.525			
Stroke	**1.4**	1.0–2.0	**0.045**	1.5	0.9–2.4	0.058
Porcelain aorta	1.4	0.7–2.6	0.325			
CAD	1.1	0.9–1.5	0.380			
Euroscore II > 5 points	**1.5**	1.1–1.9	**0.007**	1.0	0.7–1.5	0.708
NT‐proBNP ≥ 1532 ng/L	**1.7**	1.2–2.2	**0.001**	1.2	0.8–1.8	0.165
Troponin > 24 ng/mL	**2.3**	1.6–3.3	**< 0.001**	1.7	1.2–2.6	0.004
Creatinine ≥ 1.1 mg/dL	**1.5**	1.1–1.9	**0.007**			
Mean gradient > 43 mmHg	**0.7**	0.5–0.9	**0.002**	0.8	0.5–1.1	0.307
EF > 60%	**0.6**	0.5–0.8	**< 0.001**	0.8	0.6–1.2	0.539
AVA < 0.7 cm^2^	1.1	0.9–1.5	0.357			
AF	**1.4**	1.1–1.8	**0.015**	1.0	0.7–1.4	0.681
LBBB	1.4	0.9–2.0	0.119			
RBBB	1.3	0.8–2.1	0.236			
Balloon pre‐dilatation	0.8	0.2–2.3	0.622			
Balloon post‐dilatation	0.7	0.3–1.4	0.318			
RapidPacing	0.7	0.3–1.8	0.480			
Prosthesis size > 26 mm	**1.3**	1.0–1.7	**0.043**	**1.4**	1.0–2.1	**0.021**
Stroke post TAVI	**3.0**	1.3–6.8	**0.007**	1.6	0.5–5.3	0.418

*Note:* This table displays univariable and multivariable predictors of long‐term mortality in women and men. Significant variables of the univariate analysis were included in the multivariate analysis. Bold levels are significant.

Abbreviations: AF, atrial fibrillation; CAD, Coronary artery disease; CI, confidence interval; CKD, chronic kidney disease; DM, diabetes mellitus; HR, hazard ratio; LAH, left anterior hemiblock; LBBB, Left bundle branch block; LVEF, left ventricular ejection fraction; NT‐proBNP, N‐terminal pro‐brain natriuretic peptide; PAD, peripheral artery disease; PM, pacemaker; RBBB, Right bundle branch block.

#### 3.3.1 Women: Early Procedural Vulnerability Without Long‐Term Impact

**FIGURE 1:**
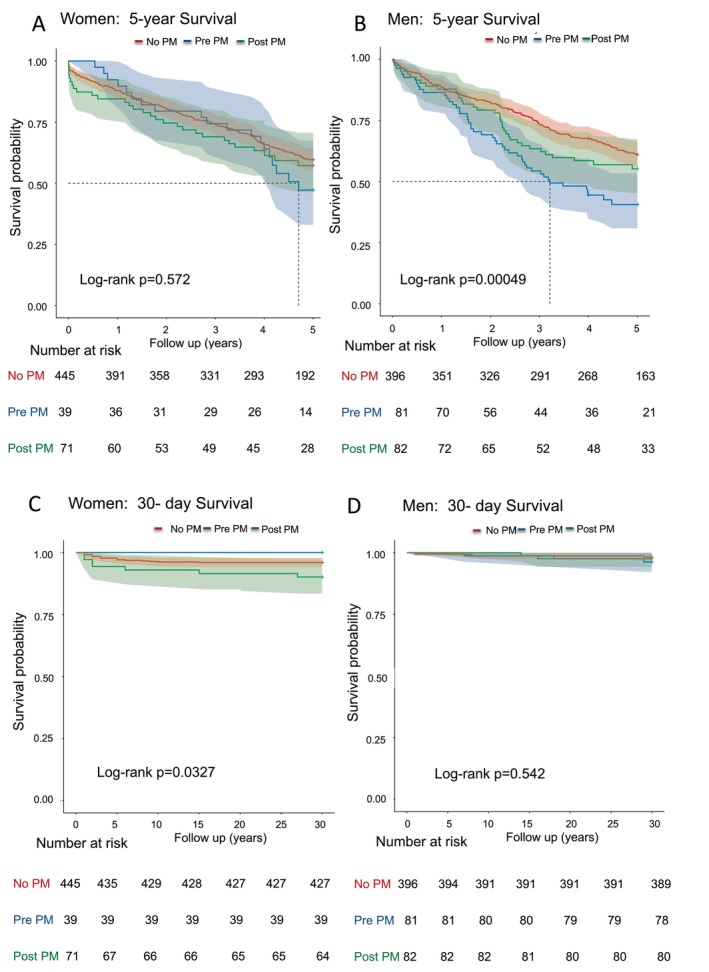
Five‐year and 30‐day survival according to PM status stratified by sex. In women, 5‐year survival did not differ significantly between patients without a PM, with a pre‐existing PM, or with a post‐procedural PM (log‐rank *p* = 0.572) (A). In men, 5‐year survival differed significantly by PM status, with the lowest survival in those with a pre‐existing PM and intermediate outcomes in those with a post‐procedural PM (log‐rank *p* = 0.00049) (B). In women, 30‐day survival differed modestly by PM status, with slightly lower early survival in the post‐procedural PM group (log‐rank *p* = 0.0327) (C). In men, 30‐day survival was uniformly high and did not differ significantly across PM groups (log‐rank *p* = 0.542) (D).

In women, post‐PM status was associated with early but not late mortality. Kaplan–Meier analysis revealed higher 30‐day mortality in the post‐PM group compared with no‐PM and pre‐PM patients (log‐rank *p* = 0.033), whereas long‐term survival remained similar across groups (log‐rank *p* = 0.572; Figure 1A,C).

Multivariable Cox regression identified age, CKD, NT‐proBNP, troponin T, mean gradient and AF as independent predictors of 5‐year mortality, while neither pre‐PM nor post‐PM status was significantly associated with prognosis.

#### 3.3.2 Men: Chronic Conduction Disease as Long‐Term Risk Marker

In men, there was no statistical significance in 30‐day survival across PM groups (log‐rank *p* = 0.542). In contrast, 5‐year survival differed markedly among PM groups (log‐rank *p* < 0.001; Figure 1B,D). Pre‐PM patients showed the lowest survival, post‐PM demonstrated an intermediate trajectory and no‐PM the most favourable course.

Multivariable Cox regression confirmed pre‐PM status as an independent predictor of 5‐year mortality, while post‐PM showed no prognostic impact. Additional independent predictors included CKD, troponin T, AVA < 0.7 cm^2^ and prosthesis size > 26 mm.

## 4 Discussion

Sex‐specific differences in TAVI outcomes are well known, yet the prognostic implications of PM status have not been examined separately in women and men. This gap is particularly relevant as TAVI expands to younger patients with longer life expectancy and prolonged exposure to conduction disease and pacing [1].

In this large cohort with long‐term follow‐up, we observed distinct sex‐specific patterns. In women, post‐PM status was associated with worse 30‐day survival but not with long‐term mortality, whereas in men, pre‐PM status emerged as the strongest independent predictor of 5‐year mortality.

The distribution of PM status differed markedly between women and men, reflecting anatomical and electrophysiological differences. Women more often presented without a pre‐existing PM, whereas men more frequently had a pre‐PM status, including ICDs and CRT‐Ds, consistent with a higher burden of baseline conduction disease. Importantly, ICD and CRT devices may also reflect underlying ventricular dysfunction and should therefore not be regarded exclusively as markers of conduction abnormalities. We therefore excluded these patients from further analysis [15].

In contrast to these baseline differences, rates of new post‐procedural PM implantation were similar across sexes, with a slight numerical increase in men. However, a large meta‐analysis, including more than 70,000 patients, reported a 10% lower risk for PM implantation after TAVI in women, independent of age or ventricular function. Notably, the authors reported that an increased use of BEVs in women attenuated the protective effect of female sex with regard to post‐procedural PM implantation [11]. These findings may explain the absence of a sex‐specific difference in our cohort, in which BEVs predominated.

In women, post‐PM status was associated with impaired 30‐day survival, suggesting increased procedural vulnerability related to new conduction disturbances and PM implantation rather than a sustained adverse prognostic effect. Supporting this concept, women in our cohort experienced significantly more major bleeding complications than men and showed numerically higher rates of stroke, cardiac tamponade and cardiopulmonary resuscitation.

Similarly, Nowak et al. reported that women undergoing PM implantation, independent of TAVI, more frequently experienced procedural complications, such as pocket haematoma, cardiac perforation and pneumothorax [16]. Anatomical differences related to smaller body size, including reduced vessel diameters, have been proposed as potential contributors to the higher incidence of complications among women [17]. In contemporary practice, the increasing use of leadless PM systems after TAVI, which are associated with lower overall complication rates than conventional transvenous devices due to the elimination of lead‐ and pocket‐related issues, may mitigate the early postoperative disadvantage associated with PM implantation in women [18, 19].

These findings are consistent with previous sex‐specific TAVI studies reporting higher rates of early procedural complications in women despite favourable long‐term outcomes [20, 21, 22, 23] Importantly, prior cohorts and our own include patients treated in earlier TAVI eras, when the 2017 ESC/EACTS guidelines recommended dual antiplatelet therapy for 3–6 months after TAVI, a practice that may have contributed to higher overall bleeding rates particularly among women [24, 25].

In men, 30‐day outcomes were similar across PM groups; however, at 5‐year follow‐up, a graded survival disadvantage emerged exclusively among male PM carriers. Survival was lowest in patients with a pre‐existing PM, intermediate in those receiving a post‐procedural PM, and most favourable in men without PM implantation. This pattern is consistent with previous reports of impaired long‐term survival among PM carriers after TAVI, although prior studies did not perform sex‐stratified analyses [2, 10, 26, 27]. Importantly, the adverse long‐term prognosis in men appears to be driven primarily by a pre‐existing PM status, which likely reflects a higher burden of baseline cardiac disease [28]. Accordingly, pre‐existing PM status should be interpreted primarily as a marker of a more advanced cardiovascular phenotype rather than a direct causal determinant of adverse long‐term prognosis.

This single‐center post hoc analysis has inherent limitations related to its observational design. Despite multivariable adjustment, residual confounding cannot be excluded, and the observed associations should therefore be interpreted with caution. Data on pacing burden, pacemaker dependency, pacing mode and right ventricular pacing percentage during follow‐up were unavailable, precluding assessment of their potential contribution to long‐term outcomes. This is particularly relevant because emerging evidence suggests that the clinical impact of pacemaker implantation after TAVI may depend on the degree of ventricular pacing, with higher right ventricular pacing percentages potentially affecting left ventricular function and long‐term prognosis. Furthermore, unmeasured procedural factors such as implantation depth, membranous septum length, valve oversizing and calcium distribution may have influenced PM implantation and clinical outcomes and could not be assessed in the present study.

Moreover, dichotomization of continuous variables may reduce statistical power and obscure non‐linear associations. However, this approach was considered appropriate to improve model stability, reduce the risk of overfitting, and facilitate interpretation of sex‐specific risk estimates in a cohort with limited numbers of events within individual subgroups.

Subgroup sizes, particularly pre‐PM in women, were small, potentially limiting statistical power in sex‐stratified analyses. Thirty‐day classification was chosen according to established VARC definitions; delayed manifestations of TAVI‐related conduction disturbances resulting in PM implantation beyond 30 days cannot be completely excluded.

The cohort predominantly included balloon‐expandable Edwards SAPIEN valves; therefore, results may not be fully generalizable to self‐expanding systems, which are associated with higher PM rates and potentially different sex‐specific outcomes. Additionally, this study represents a decade of TAVI practice and therefore includes patients treated across different procedural eras, incorporating both earlier and contemporary technologies as well as evolving operator experience, largely preceding the widespread adoption of newer‐generation devices. Finally, systematic data on heart failure hospitalizations and cause‐specific mortality were not available, precluding assessment of cardiovascular versus non‐cardiovascular causes of death.

## 5 Conclusions

Women exhibit greater early procedural vulnerability without clear evidence of a sustained adverse prognostic impact, whereas in men pre‐existing conduction disease appears to be more closely associated with long‐term mortality. These findings highlight the potential value of sex‐stratified risk assessment, follow‐up planning and suggest that future risk models may need to consider PM status differently in women and men.

## Author Contributions

C.V. and G.L. contributed to conceptualization, methodology, investigation, writing – original draft, project administration. M.W., M.H., M.G., L.M. and K.S. performed data curation, investigation, writing – review and editing. J.M.: conceptualization, resources, writing – review and editing, supervision. All authors read and approved the final manuscript.

## Funding

The authors have nothing to report.

## Disclosure

The authors have nothing to report.

## Conflicts of Interest

The authors declare no conflicts of interest.

## Supporting information


**Table S1:** Individual causes of 30‐day mortality among women with new pacemaker implantation (*n* = 7).

## Data Availability

All data generated or analysed during this study are included in this published article.
