# Supplementary material for: Pacemaker Status and 5‐Year Mortality After TAVI: A Sex‐Specific Analysis
Source: Eur J Clin Invest. 2026 Jul 2;56(7):e70242. doi: 10.1111/eci.70242 (PMC13329084; doi:10.1111/eci.70242)
Supplement: Supplementary file 1 — Table S1: Individual causes of 30‐day mortality among women with new pacemaker implantation (n = 7). [file ECI-56-e70242-s001.docx]

Supplementary Material

Pacemaker Status and 5-Year Mortality After TAVI: A Sex-Specific Analysis

**Running title:** Sex-Specific Impact of PM After TAVI

Cecilia Veraar, MD^1*^, Gudrun Lamm, MD^1*^ Maximilian Will, MD^1^; Matthias Hammerer^2^, Matthias Granner^1^, Lion Merl^1^, Konstantin Schwarz, MD^1^; Julia Mascherbauer, MD^1^

* These authors contributed equally to this work and share first authorship.

**Affiliations:**

^1^ Department of Internal Medicine 3, University Hospital St. Pölten – NOE LGA, Karl Landsteiner University, Dunant-Platz 1, 3100, St. Pölten, Austria

^2^ Department of Internal Medicine II, Paracelsus Medical University of Salzburg,

Salzburg, Austria

**Supplementary Table 1:** Individual Causes of 30-Day Mortality Among Women With New Pacemaker Implantation (n = 7)

| 1. | Complete AV block requiring permanent pacemaker implantation. The procedure was complicated by pericardial effusion with cardiac tamponade requiring cardiopulmonary resuscitation, followed by pulmonary hemorrhage. The patient subsequently died from refractory hypovolemic shock. |
| --- | --- |
| 2. | Fatal procedural complication: left main coronary artery occlusion during TAVI requiring CPR and emergency left main PCI, followed by complete AV block requiring pacemaker implantation. Recurrent cardiac arrest occurred during pacemaker implantation, resulting in death. |
| 3. | Refractory cardiogenic and circulatory failure following pericardial tamponade due to ventricular perforation after pacemaker implantation, despite pericardiocentesis and surgical repair. |
| 4. | Hypovolemic shock secondary to pulmonary artery bleeding causing hemothorax after pacemaker implantation; |
| 5. | Fatal pulmonary hemorrhage following pacemaker implantation for complete AV block, resulting in hypovolemic shock and cardiac arrest. |
| 6. | Complete AV block requiring permanent pacemaker implantation. The post-procedural course was complicated by pericardial effusion requiring drainage and cardiopulmonary resuscitation, followed by intensive care treatment. Progressive renal and respiratory failure resulted in multiorgan failure and death. |
| 7. | Periprocedural stroke during TAVI requiring endotracheal intubation. The subsequent clinical course was complicated by progressive multiorgan failure, resulting in death. |
